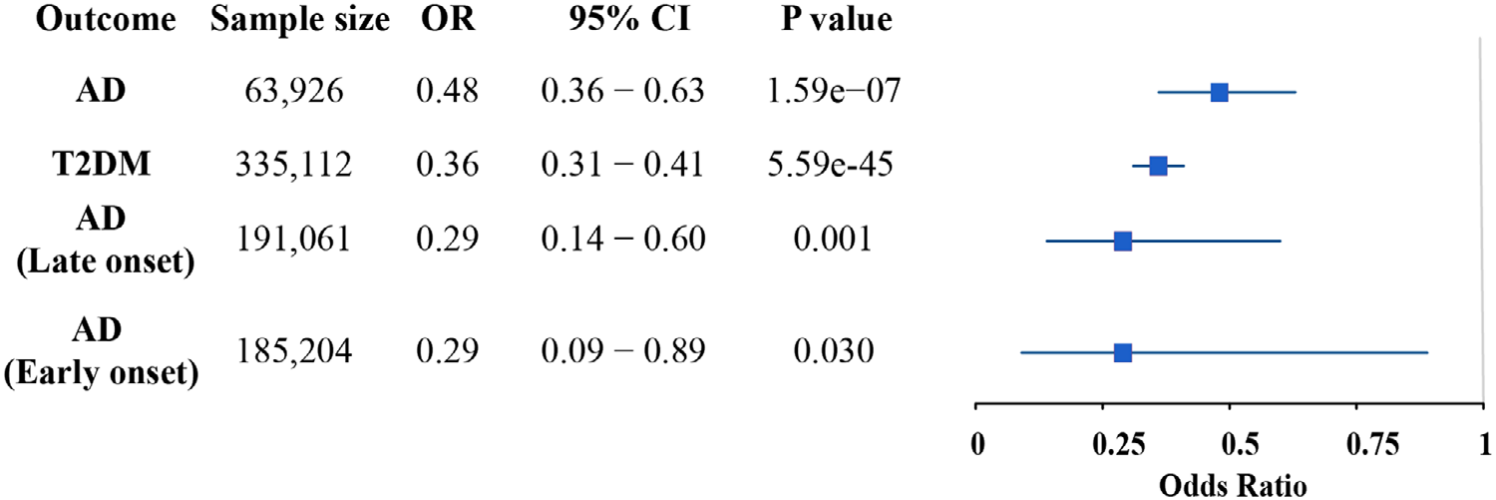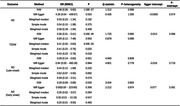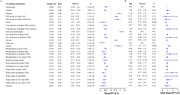# Unraveling the Link Between SGLT2 Inhibition, Circulating Metabolites, and Alzheimer’s Dementia: A Mendelian Randomization Study

**DOI:** 10.1002/alz.089014

**Published:** 2025-01-09

**Authors:** Hao Yang, Yuye Ning, Meilin Chen, Jianping Jia

**Affiliations:** ^1^ Xuanwu hospital Capital Medical University, Beijing, Beijing China; ^2^ Xuanwu Hospital, Capital Medical University, Beijing, Beijing China; ^3^ Innovation Center for Neurological Disorders, Xuanwu Hospital, Capital Medical University, Beijing, China;, Beijing China

## Abstract

**Background:**

Individuals with type 2 diabetes mellitus (T2DM) face an increased risk of dementia. Recent discoveries indicate that SGLT2 inhibitors, a newer class of anti‐diabetic medication, exhibit beneficial metabolic effects beyond glucose control, offering a potential avenue for mitigating the risk of Alzheimer’s disease (AD). However, limited evidence exists regarding whether the use of SGLT2 inhibitors effectively reduces the risk of AD. Previous cohort studies encountered challenges related to potential reverse causality. Hence, our study aims to explore the impact of circulating metabolites mediating SGLT2 inhibition on AD through Mendelian randomization (MR).

**Method:**

In this study, a two‐sample Mendelian randomization (MR) approach was employed to investigate the association between SGLT2 inhibition and AD. Genetic instruments for SGLT2 inhibition were identified based on variants associated with the expression of the SLC5A2 gene and glycated hemoglobin level (HbA1c). Positive control analysis for T2DM validated the chosen genetic instruments. Furthermore, we explored the relationships between SGLT2 inhibition and different forms of AD (late‐onset and early‐onset AD). Two‐step MR was utilized to examine the mediation effects of circulating metabolites linking SGLT2 inhibition with AD.

**Result:**

Genetically predicted SGLT2 inhibition (per 1 SD decrement in HbA1c) was associated with a reduced risk of both T2DM (odds ratio [OR] = 0.36 [95% CI 0.31, 0.41], p < 0.001) and AD (0.48 [0.36, 0.63], p < 0.001). The causal relationship between SGLT2 and late‐onset and early‐onset AD was consistent (OR = 0.29 [0.14‐0.60], p = 0.001; 0.29 [0.09‐0.89], p = 0.03). Among the 123 circulating metabolites examined, citrate demonstrated significant associations with both SGLT2 inhibition and AD (0.81 [0.79, 0.83, p < 0.001]).

**Conclusion:**

This study provides robust evidence supporting the association between SGLT2 inhibition and a reduced risk of AD. Notably, citrate levels may mediate this association, emphasizing the imperative for further investigation through randomized controlled trials.